# 

**Published:** 1875-12

**Authors:** 


FOUR DOLLARS A YEAR, POSTAGE FREE.

VOL. XXXII. December, 1875. NUMBER 12.

THE

### Chicago Medical Journal

AND

### EXAMINER.

ISSUED TWELVE TIMES A YEAR.

FOLIA SUA AD SANITATEM GENTIUM

**EDITOR:**

WILLIAM H. BYFORD, A.M., M.D.

**ASSOCIATE EDITORS:**

JAS. H. ETHERIDGE, M.D.

NORMAN BRIDGE, M.D.

JAS. NEVINS HYDE, A.M., M.D.

FERD. C. HOTZ, M.D.

Published under the auspices of the

*CHICAGO MEDICAL PRESS ASSOCIATION.*

TERMS—FOUR DOLLARS PER ANNUM, IN ADVANCE.

CHICAGO:

W. B. KEEN, COOKE & CO., Publishers,

NOS. 113 AND 115 STATE STREET.

